# Supplementary figures and images for: Genome-Wide Identification and Expression Profiling of Monosaccharide Transporter Genes Associated with High Harvest Index Values in Rapeseed (Brassica napus L.)
Source: Genes (Basel). 2020 Jun 15;11(6):653. doi: 10.3390/genes11060653 (PMC7349323; doi:10.3390/genes11060653)

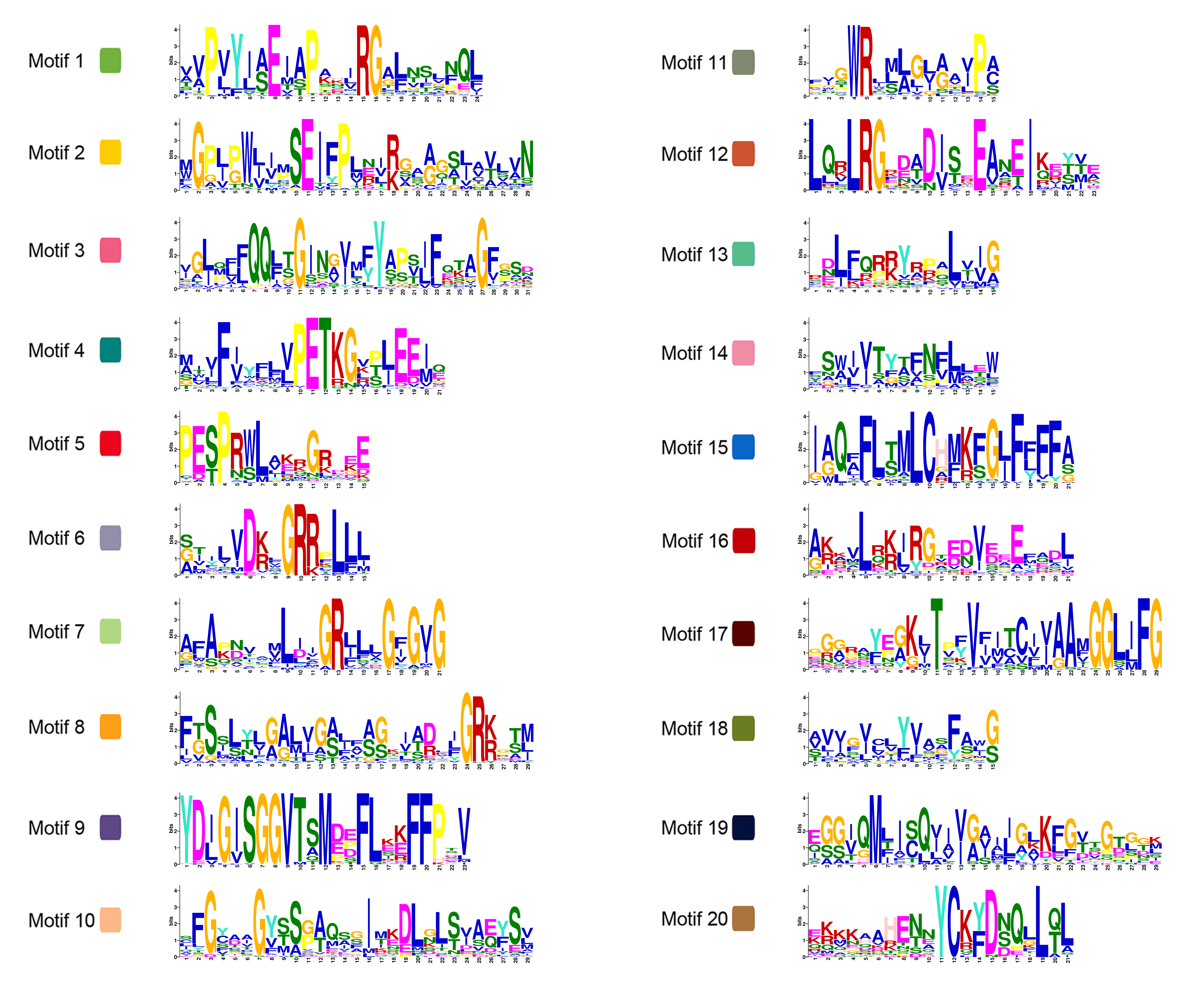

Supplement: Supplementary file 1 [file genes-11-00653-s001.zip › Supplementary/Figure S1.jpg]

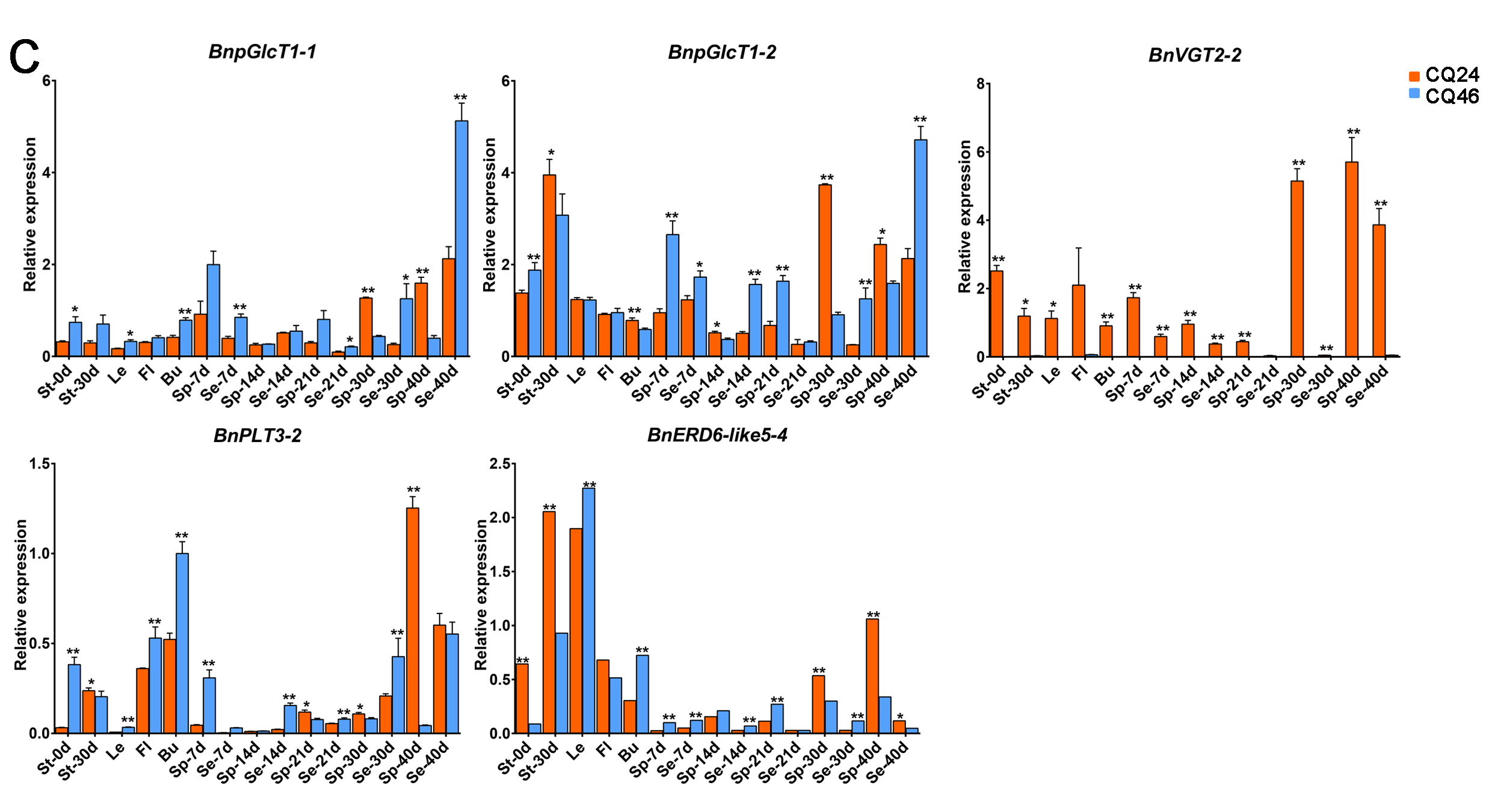

Supplement: Supplementary file 1 [file genes-11-00653-s001.zip › Supplementary/Figure S2.jpg]
